# Impact of Intravenous Alteplase Door-to-Needle Times on 2-Year Mortality in Patients With Acute Ischemic Stroke

**DOI:** 10.3389/fneur.2021.747185

**Published:** 2021-10-13

**Authors:** Nirav R. Bhatt, Anika Backster, Moges S. Ido, Raul G. Nogueira, Rana Bayakly, David W. Wright, Michael R. Frankel

**Affiliations:** ^1^Marcus Stroke and Neuroscience Center, Grady Memorial Hospital, Department of Neurology, Emory University School of Medicine, Atlanta, GA, United States; ^2^Department of Emergency Medicine, Emory University School of Medicine, Atlanta, GA, United States; ^3^Division of Epidemiology and Biostatistics, School of Public Health, Georgia State University, Atlanta, GA, United States; ^4^Georgia Department of Public Health, Division of Health Protection, Epidemiology Program, Atlanta, GA, United States

**Keywords:** acute stroke care, tissue plasminogen activator, mortality, door to needle time, thrombolytic therapy

## Abstract

**Objective:** We sought to determine whether administration of Intravenous Thrombolysis (IVT) to patients with Acute Ischemic Stroke (AIS) within 60 min from hospital arrival is associated with lower 2-year mortality.

**Methods:** This retrospective study was conducted among patients receiving IVT in hospitals participating in the Georgia Coverdell Acute Stroke Registry (GCASR) from January 1, 2008 through June 30, 2018. Two-year mortality data was obtained by linking the 2008–2018 Georgia Discharge Data System data and the 2008–2020 Georgia death records. We analyzed the study population in two groups based on the time from hospital arrival to initiation of IVT expressed as Door to Needle time (DTN) in a dichotomized (DTN ≤ 60 vs. > 60 min) fashion.

**Results:** The median age of patients was 68 years, 49.4% were females, and the median NIHSS was 9. DTN ≤60 min was associated with lower 30-day [odds ratio (OR), 0.62; 95% CI, 0.52–0.73; *P* < 0.0001], 1-year (OR, 0.71; 95% CI, 0.61–0.83; *P* < 0.0001) and 2-year (OR, 0.76; 95% CI, 0.65–0.88; *P* = 0.001) mortality as well as lower rates of sICH at 36 h (OR, 0.57; 95% CI, 0.43–0.75; *P* = 0.0001), higher rates of ambulation at discharge (OR, 1.38; 95% CI, 1.25–1.53; *P* < 0.0001) and discharge to home (OR, 1.36; 95% CI, 1.23–1.52; *P* < 0.0001).

**Conclusion:** Faster DTN in patients with AIS was associated with lower 2-year mortality across all age, gender and race subgroups. These findings reinforce the need for intensifying quality improvement measures to reduce DTN in AIS patients.

## Introduction

Stroke is the fifth leading cause of death in the United States (US) (1, 2). The south-eastern states have the unenviable distinction of being the “Stroke-belt,” accounting for higher stroke mortality as compared to rest of the US (3). Even worse, regions within the “Stroke-belt” states, including parts of Georgia along with North and South Carolina, form what is known as the “Stroke-buckle” where Stroke mortality is ~40% higher than rest of the states in the US (4). Although randomized clinical trials of IVT in patients with AIS have shown a time-sensitive beneficial reduction in long-term disability, there was no reduction in mortality at 3 months or at 1 year, possibly due to a small but clinically relevant increased risk of fatal intracranial hemorrhage (5–7). Although an analysis of the Get With The Guidelines-Stroke (GWTG) database provided clarity on the beneficial relationship between earlier IVT and short-term mortality, leading to further refinements of the consensus guidelines emphasizing the importance of time to IVT, the durability of this time-sensitive benefit on longer-term mortality remains to be determined (8). A more recent GWTG based study of Medicare patients showed beneficial effects of faster IVT administration and 1-year mortality (9). However, limitations of the study included under-representation of racial minorities and only including older, Medicare population. Thus, a better understanding of longer-term outcome across broader age and racial groups is needed. Our study aims to investigate the effect of faster treatment with IVT on long-term mortality among AIS patients in the GCASR.

## Methods

### Study Design

We conducted a retrospective cohort study on AIS patients treated with IVT at hospitals participating in the GCASR. As part of the Centers for Disease Control and Prevention (CDC) National Paul Coverdell Acute Stroke Program initiative (10), GCASR aims to improve the quality of stroke care across the state of Georgia. The Emory University institutional review board approved the study and as it met the exceptions for informed consent requirements, the need for informed consent was waived. This study adheres to the Strengthening the Reporting of Observational Studies in Epidemiology (STROBE) guideline.

### Data Sources

We used three data sources: GCASR data, the Georgia Discharge Data System (GDDS), and Georgia death data. The GCASR and GDDS data were linked using a hierarchical deterministic linkage procedure (sensitivity 87%, positive predictive value 96%) (11); the output was then linked with the death data applying a probabilistic linkage approach (sensitivity 92%, specificity 100%) (12). The probabilistic linkage procedure and its yield have been described previously (13).

### Study Population

**Figure 1:**
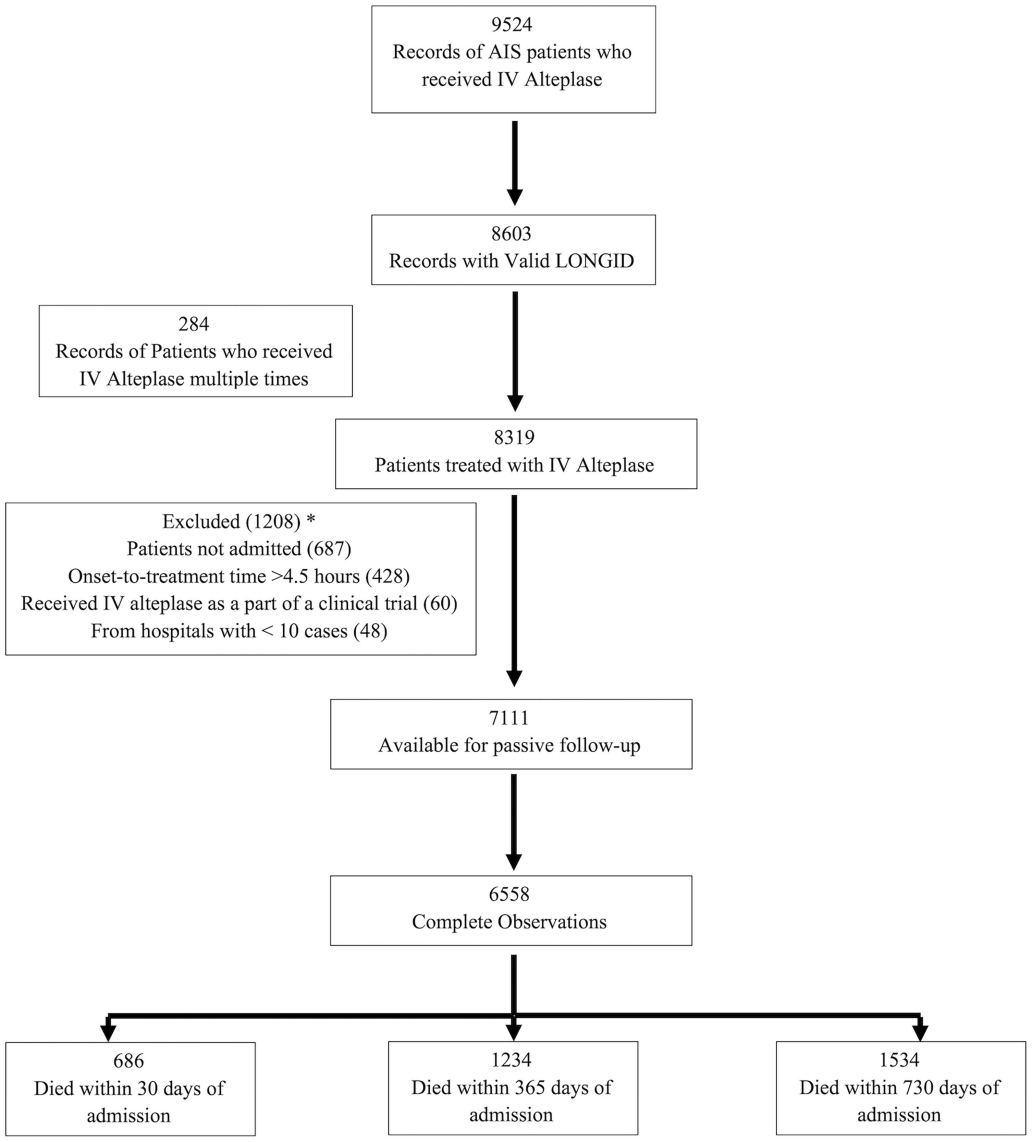
Flow chart for the number of subjects included in the analyzes. *Some patients had >1 reason for exclusion.

A total of 9,524 records of adult AIS patients treated with IVT were captured by the registry over the period from January 1, 2008–June 30, 2018. Among these, 8,603 had valid linkage information. As the intent of this analysis was to focus on determining the relationship between time and outcome in patients who were treated with standard IVT in the emergency department, we excluded 1,492 due to in-hospital stroke, those receiving IVT as part of a clinical trial or outside the 4.5 h treatment window, or those who were either not admitted or received IVT more than once (Figure 1).

### Outcome Measures and Predictors

The primary outcome was death from any cause at 30, 365, and 730 days after admission. We considered patients to be alive if neither the hospital discharge data reported them as having “Expired” under final discharge disposition nor were captured in the Georgia state death record. For secondary outcomes, we analyzed patients' ambulatory status at discharge (among those who were ambulating independently prior to admission), patients' discharge destination, and whether they developed symptomatic intracranial hemorrhage (sICH) within 36 h of IVT treatment.

Door-to-needle time (DTN), the primary predictor, was defined as the time difference between patient arrival at a hospital and the initiation of IVT. Based on a previous publication on predictors of in-hospital death (8) and clinical experience, we considered the following variables as covariates: socio-demographic characteristics (age, sex, and race), hospital bed-size, event-related characteristics (last known well to hospital arrival time, mode of transport to hospital, and calendar year), National Institutes of Health Stroke Scale (NIHSS) score, and patient medical history (atrial fibrillation/flutter, coronary artery disease/prior myocardial infarction, diabetes mellitus, dyslipidemia, heart failure, hypertension, smoking, previous stroke or transient ischemic attack).

### Statistical Analysis

DTN was categorized into ≤60 and >60 min categories based on commonly held clinical intervals of significance and promoted by the quality improvement activities of the GCASR. Descriptive statistics were used to compare patient characteristics by DTN time. We assessed the association between DTN and the primary (30-day, 1-year, and 2-year mortality) and secondary outcomes using generalized estimating equations (GLIMMIX procedure) controlling for confounders and in-hospital correlation and considering hospital as a random variable. To assess effect modification by age, gender and race, we included interaction terms between DTN time and socio-demographic characteristics in the regression model. To evaluate the role of Intra-arterial (IA) Alteplase or mechanical thrombectomy as potential confounders, we conducted a separate analysis excluding the patients receiving IA alteplase/ MER and assessed the association between DTN and primary outcome. Age and last known well to hospital arrival time were centered around their mean values. To maintain stable estimates, patients from hospitals with <10 cases were excluded. About 7.8% of the observations had at least one missing value, mainly the NIHSS, and they were excluded from the multivariable regressions assuming a general missing pattern and values were missing at random. All Analyses were performed using SAS version 9.4 (SAS Institute Inc., Cary, NC, USA).

### Data Availability Policy

The data that support the findings of this study are available from the corresponding author upon reasonable request.

## Results

A total of 6,558 patients with ischemic stroke received IVT and had complete information available for passive follow-up of mortality. Of these, 686 patients died at 30 days, 1,234 by 365 days and 1,534 by 730 days (Figure 1). Age ranged between 18 and 103 with a median of 68 years, 49.4% were female, and 35.5% were black. Median time from last known well to hospital arrival was higher in the ≤ 60 min DTN group [68 (IQR, 45–106) min] as compared to the > 60 min DTN group [60 (IQR, 41–91) min]. The median NIHSS was 9 for both groups. There were fewer patients with history of atrial fibrillation and prior stroke or TIA in the ≤ 60 min group as compared to the > 60 min group (Table 1).

**Table 1:** Characteristics and outcomes of ischemic stroke patients treated with intravenous alteplase, GCASR, January 2008–June 2018.

**Characteristic/outcomes**	**Total**	**Door-to-IV alteplase time**		***P*-value^a^**
		**≤60 min (*n* = 3,722)**	**>60 min (*n* = 2,836)**	
Age, years, median (IQR)	68 (56, 79)	68 (57, 78)	68 (56, 79)	0.91
Female, *n* (%)	3,241 (49.4)	1,722 (46.3)	1,519 (53.6)	<0.0001
**Race**, ***n*** **(%)**				
Whites	4,125 (62.9)	2,363 (63.5)	1,762 (62.1)	0.53
Blacks	2,327 (35.5)	1,300 (34.9)	1,027 (36.2)	
Others	106 (1.6)	59 (1.6)	47 (1.7)	
NIH Stroke scale score, unit, median (IQR)	9 (5, 16)	9 (5, 15)	9 (5, 16)	0.05
**Previous medical history of**, ***n*** **(%)**				
Atrial fibrillation or flutter	1,115 (17.0)	595 (16.0)	520 (18.3)	0.01
Dyslipidemia	2,640 (40.3)	1,527 (41.0)	1,113 (39.2)	0.15
Diabetes mellitus	1,838 (28.0)	1,029 (27.6)	809 (28.5)	0.43
History of heart failure	724 (11.0)	411 (11.0)	313 (11.0)	0.99
Hypertension	5,027 (76.7)	2,854 (76.7)	2,173 (76.6)	0.96
Coronary artery disease/prior MI	1,496 (22.8)	834 (22.4)	662 (23.3)	0.37
Smoking in the past 1 year	1,506 (23.0)	856 (23.0)	650 (22.9)	0.94
Stroke or transient ischemic attack	1,694 (25.8)	916 (24.6)	778 (27.4)	0.01
Last known well to hospital arrival time, minutes, median (IQR)	64 (43, 100)	68 (45, 106)	60 (41, 91)	<0.0001
**Brought to hospital**				
Emergency medical service	5,267 (80.3)	3,076 (82.6)	2,191 (77.3)	<0.0001
Private transport	1,200 (18.3)	584 (15.7)	616 (21.7)	
Transferred from other hospital	91 (1.4)	62 (1.7)	29 (1.0)	
**Hospital bed size**, ***n*** **(%)**				
<500 beds	3,496 (53.3)	1,836 (49.3)	1,660 (58.5)	<0.0001
500+ beds	3,062 (46.7)	1,886 (50.7)	1,176 (41.5)	
**Death**, ***n*** **(%)**				
Death in 30 days	686 (10.5)	319 (8.6)	367 (12.9)	<0.0001
Death in 1-year	1,234 (18.8)	622 (16.7)	612 (21.6)	<0.0001
Death in 2-year	1,534 (23.4)	795 (21.4)	739 (26.1)	<0.0001
Ambulate independently at discharge,^b^ *n* (%)	3,223 (53.2)	1,964 (56.3)	1,259 (49.1)	<0.0001
Intracranial hemorrhage within 36 h, *n* (%)	230 (3.7)	97 (2.7)	133 (5.0)	<0.0001
**Discharged disposition**, ***n*** **(%)**				
Home	3,453 (53.0)	2,052 (55.6)	1,401 (49.7)	<0.0001
Healthcare facilities but not hospice	2,359 (36.2)	1,305 (35.4)	1,054 (37.4)	
Hospice or died	697 (10.7)	333 (9.0)	364 (12.9)	

*IQR, Interquartile range*.

a *χ2 and Wilcoxon tests were applied for nominal and quantitative variables, respectively*.

b *Able to ambulate independently with or without a device but no assistance from another person among patients who were ambulating on admission*.

Patients who received IVT ≤60 min from hospital arrival had a 38% [OR 0.62 (95% CI, 0.52–0.73)], 29% [OR 0.71 (95% CI, 0.61–0.83)] and 24% [OR 0.76 (95% CI, 0.65–0.88)] risk reduction in 30-day, 1-year, and 2-year mortality, respectively, compared to those who were treated after 60 min of hospital arrival (**Table 3**). As age and NIHSS increased, the relative risk of mortality at 30 days, 1 year, and 2 year increased (Table 2). History of atrial fibrillation and diabetes mellitus were also associated with higher 30-day, 1-year, and 2-year mortality. The interaction terms between DTN and socio-demographic characteristics—age, gender and race group—were not statistically significant and were not included in the models.

**Table 2:** Relative risk of mortality among acute ischemic stroke patients treated with intravenous alteplase.

**Predictors**	**30-day**^**a**^ **mortality**		**1-yr**^**a**^ **mortality**		**2-yr**^**a**^ **mortality**	
	**Odds ratio^**b**^ (95% CL)**	***P-*value**	**Odds ratio^**b**^ (95% CL)**	***P-*value**	**Odds ratio^**b**^ (95% CL)**	***P-*value**
**Age groups**						
>80 years	6.22 (4.34, 8.92)	<0.0001	11.27 (8.78, 14.47)	<0.0001	12.06 (9.80, 14.84)	<0.0001
66–80 years	2.16 (1.51, 3.10)		3.89 (3.05, 4.95)		3.86 (3.18, 4.68)	
56–65 years	1.61 (1.06, 2.44)		2.01 (1.50, 2.68)		1.98 (1.53, 2.55)	
≤ 55 years	Referent		Referent		Referent	
**Gender**						
Female	0.92 (0.79, 1.06)	0.24	0.95 (0.80, 1.12)	0.51	0.92 (0.80, 1.06)	0.24
Male	Referent		Referent		Referent	
**Race**						
Others	1.11 (0.43, 2.82)	0.01	0.96 (0.48, 1.91)	0.94	0.88 (0.49, 1.59)	0.43
Blacks	0.75 (0.63, 0.90)		1.02 (0.90, 1.16)		1.07 (0.94, 1.22)	
Whites	Referent		Referent		Referent	
**NIH stroke scale score (unit)**						
>15	9.24 (6.21, 13.75)	<0.0001	5.37 (4.24, 6.80)	<0.0001	4.49 (3.35, 6.03)	<0.0001
11–15	4.28 (2.93, 6.25)		2.69 (2.19, 3.30)		2.55 (2.05, 3.17)	
6–10	2.24 (1.47, 3.43)		1.53 (1.20, 1.94)		1.49 (1.17, 1.88)	
≤ 5	Referent		Referent		Referent	
**Previous medical history of**						
Atrial fibrillation or flutter	1.79 (1.46, 2.19)	<0.0001	1.62 (1.38, 1.89)	<0.0001	1.58 (1.35, 1.86)	<0.0001
Dyslipidemia	0.91 (0.75, 1.10)	0.31	0.77 (0.66, 0.90)	0.002	0.76 (0.67, 0.87)	0.0001
Diabetes mellitus	1.37 (1.09, 1.72)	0.01	1.39 (1.13, 1.70)	0.002	1.36 (1.13, 1.63)	0.002
History of heart failure	1.09 (0.83, 1.43)	0.55	1.51 (1.24, 1.84)	0.0001	1.70 (1.38, 2.10)	<0.0001
Hypertension	1.31 (0.98, 1.75)	0.07	1.27 (1.07, 1.52)	0.01	1.41 (1.17, 1.71)	0.001
Coronary artery disease/prior MI	1.15 (0.94, 1.42)	0.18	1.21 (1.06, 1.38)	0.01	1.27 (1.09, 1.48)	0.003
Smoking in the past 1 year	1.26 (1.00, 1.57)	0.05	1.22 (1.01, 1.46)	0.04	1.13 (0.95, 1.34)	0.16
Stroke or transient ischemic attack	0.86 (0.71, 1.04)	0.12	1.02 (0.83, 1.26)	0.84	1.14 (0.95, 1.37)	0.15
**Brought to hospital by**						
Transferred from other hospital	0.88 (0.42, 1.83)	0.002	0.78 (0.47, 1.32)	0.003	0.73 (0.42, 1.29)	0.002
Private transport	0.51 (0.35, 0.74)		0.59 (0.43, 0.79)		0.61 (0.46, 0.80)	
Emergency medical service	Referent		Referent		Referent	
**Bed size**						
500+ beds	0.79 (0.63, 0.99)	0.04	0.83 (0.69, 0.99)	0.04	0.87 (0.73, 1.04)	0.11
<500 beds	Referent		Referent		Referent	

*95%CL, 95% confidence limit*.

a *Days are counted from admission date*.

b *Estimates are adjusted for calendar year and last known well to hospital arrival time*.

The frequency and adjusted risk for sICH was lower in the group receiving IVT < 60 min compared to those receiving IVT after 60 min [2.7 vs. 5.0%; OR, 0.5 (95% CI, 0.43–0.75)]. Similarly, patients with IVT ≤ 60 min were more likely to be ambulatory at discharge [OR, 1.38 (95% CI, 1.25–1.53)] and more likely to be discharged to home [OR, 1.36 (95% CI, 1.23–1.52)] (Table 3). In stratified analysis, patients with DTN time ≤ 60 min had significantly lower 30-day, 1-year, and 2-year mortality across all age, race and gender subgroups, with the exception of 56–65 year old patients, in whom a trend toward lower 2-year mortality was seen (Table 4).

**Table 3:** Relative risk of mortality among ischemic stroke patients treated with intravenous alteplase and secondary outcomes by DTN.

**Primary outcomes**						
**Door-to-needle time**	**30-day**^**a**^ **mortality**		**1-yr**^**a**^ **mortality**		**2-yr**^**a**^ **mortality**	
	**Odds ratio**^**b**^ **(95% CL)**	***P*** **-value**	**Odds ratio**^**b**^ **(95% CL)**	***P-*** **value**	**Odds ratio**^**b**^ **(95% CL)**	***P-*** **value**
≤ 60 min	0.62 (0.52, 0.73)	<0.0001	0.71 (0.61, 0.83)	<0.0001	0.76 (0.65, 0.88)	0.001
>60 min	Referent		Referent		Referent	
**Secondary outcomes**						
**Door-to-needle time**	**Intracranial hemorrhage** ** <36 h**^**c**^		**Ambulate**^**d**^ **at discharge**		**Discharge home**	
	**Odds ratio**^**b**^ **(95% CL)**	***P-*** **value**	**Odds ratio**^**b**^ **(95% CL)**	***P*** **-value**	**Odds ratio**^**b**^ **(95% CL)**	***P-*** **value**
≤60 min	0.57 (0.43, 0.75)	0.0001	1.38 (1.25, 1.53)	<0.0001	1.36 (1.23, 1.52)	<0.0001
>60 min	Referent		Referent		Referent	

*95%CL, 95% confidence limit*.

a *Days are counted from admission date*.

b *Estimates are adjusted for age, sex, race, National Institute of Health stroke scale score, previous medical illness, duration of last known well to hospital arrival time, year of admission, and hospital number of beds*.

c *Patients whose clinical condition deteriorated due to a CT detected intracranial hemorrhage within 36 h of IV alteplase administration*.

d *Ambulate independently with or without a device but no assistance from another person among patients who were ambulating on admission*.

**Table 4:** Results from stratified analyses of adjusted odds ratio.

**Category**	**30-day mortality** ^**a**^		**1-year mortality** ^**a**^		**2-year mortality** ^**a**^	
	**Odds ratio^**b**^ (95% CL)**	***P-*value**	**Odds ratio^**b**^ (95% CL)**	***P-*value**	**Odds ratio^**b**^ (95% CL)**	***P-*value**
**Age group**						
≤55 years	0.46 (0.22, 0.97)	0.04	0.52 (0.30, 0.91)	0.02	0.57 (0.38, 0.86)	0.01
56–65 years	0.53 (0.36, 0.77)	0.002	0.70 (0.50, 0.98)	0.04	0.78 (0.58, 1.04)	0.09
66–80 years	0.71 (0.47, 1.08)	0.10	0.74 (0.58, 0.95)	0.02	0.77 (0.59, 0.99)	0.04
>80 years	0.57 (0.44, 0.75)	0.0001	0.71 (0.56, 0.90)	0.01	0.76 (0.60, 0.97)	0.03
**Gender**						
Male	0.55 (0.41, 0.75)	0.0003	0.71 (0.57, 0.89)	0.004	0.75 (0.61, 0.91)	0.01
Female	0.66 (0.52, 0.84)	0.001	0.71 (0.59, 0.86)	0.001	0.76 (0.62, 0.94)	0.01
**Race group**						
Black	0.61 (0.42, 0.89)	0.01	0.70 (0.52, 0.95)	0.02	0.70 (0.53, 0.93)	0.02
White	0.66 (0.52, 0.83)	0.001	0.72 (0.59, 0.87)	0.001	0.78 (0.65, 0.93)	0.01
All subjects	0.62 (0.52, 0.73)	<0.0001	0.71 (0.61, 0.83)	<0.0001	0.76 (0.65, 0.88)	0.001

*95%CL, 95% confidence limit*.

a *Days are counted from admission date*.

b *Estimates are adjusted for age, sex, race, National Institute of Health stroke scale score, previous medical illness, duration of last known well to hospital arrival time, year of admission, and hospital number of beds*.

Similarly, when patients receiving intra-arterial Alteplase or mechanical thrombectomy were excluded, those with DTN time ≤ 60 min had lower 30-day, 1-year, and 2-year mortality as compared to those receiving IVT after 60 min (Table 5).

**Table 5:** Relative risk of mortality among ischemic stroke patients treated with intravenous alteplase, GCASR January 2008–June 2018.

**Predictors**	**30-day**^**a**^ **mortality**		**1-yr**^**a**^ **mortality**		**2-yr**^**a**^ **mortality**	
	**Odds ratio^**b**^ (95% CL)**	***P-*value**	**Odds ratio^**b**^ (95% CL)**	***P-*value**	**Odds ratio^**b**^ (95% CL)**	***P*-value**
**Patients who received IA alteplase/MER are included**						
**Door-to-needle time**						
≤ 60 min	0.62 (0.52, 0.73)	<0.0001	0.71 (0.61, 0.83)	<0.0001	0.76 (0.65, 0.88)	0.001
>60 min	Referent		Referent		Referent	
**Patients who received IA alteplase/MER are excluded**						
**Door-to-needle time**						
≤ 60 min	0.62 (0.52, 0.74)	<0.0001	0.72 (0.61, 0.85)	0.0002	0.77 (0.65, 0.90)	0.002
>60 min	Referent		Referent		Referent	

*95%CL, 95% confidence limit*.

a *Days are counted from admission date*.

b *Estimates are adjusted for age, sex, race, National Institute of Health stroke scale score, previous medical illness, duration of last known well to hospital arrival time, year of admission, and hospital number of beds*.

## Discussion

In this large, multi-center statewide registry of stroke patients treated with IVT, we found a strong and consistent association of DTN time with short- and long-term mortality. A DTN time 60 min or less was associated with 38% lower 30-day mortality, 29% lower 1-year mortality and 24% lower 2-year mortality. In addition, the risk of sICH, the most feared complication of IVT, was significantly lower in patients who were treated with IVT within 60 min of hospital arrival, and well-below published rates at 6.8% (6). Patients treated with IVT within 60 min of hospital arrival were also more likely to be ambulatory at the time of hospital discharge and more likely to be discharged home.

Despite an increased risk of sICH in patients treated with IVT, the landmark NINDS tPA trial showed no difference in 1-year mortality in AIS patients as compared to the patients who did not receive this treatment (14). Pooled analysis of individual trial data showed a modest association of earlier onset to treatment time (OTT) in reducing 90-day mortality as a continuous variable but failed to show any statistically significant mortality benefit at individual time epochs (0–90 min, 91–180 min, 181–270 min) (15). Furthermore, long-term mortality benefit with the use of IVT has been debated in several prospective observational studies (16–19). Concerns about the risk of IVT contribute to the reluctance of many clinicians to use alteplase in eligible patients despite consensus recommendations indicating Level 1A evidence.

A recent study of medicare beneficiaries showed that a shorter DTN was associated with lower 1-year mortality and reduced rates of readmission among patients 65 years and older (9). Besides the age of the population, one of the limitations of this study was related to missing Medicare claims data excluding a substantial proportion of patients leading to relative under-representation of racial minorities. Our study included all age-groups in a diverse and mostly bi-racial cohort (>30% black) that not only confirms the impact of faster treatment with IVT on reducing mortality but does so across a broader range of stroke patients while also highlighting for the first time the durability of benefit at 2 years after stroke.

Previous studies have highlighted the underutilization of IVT among black patients with AIS (20–22). However, after adjusting for IVT contraindications, the treatment rates among blacks were found to be comparable to whites, citing a delay in hospital arrival as one of the main reasons accounting for lower rates of IVT administration among blacks (22). Delays in recognition of stroke signs and underutilization of EMS among blacks have been shown to contribute to these delays (23, 24). In a GWTG-based study, although the onset-to-arrival times were similar between the blacks and whites, there was a slight delay in IVT administration among the blacks (25). While our study did not capture the reasons for delay in IVT administration, we did not find any racial disparities among the groups of patients receiving IVT within 60 min compared to those receiving IVT after 60 min of hospital arrival.

Furthermore, in our study, 30-day mortality was significantly lower in blacks as compared to whites. Similar findings have been reported for in-hospital mortality among blacks as compared to whites in previous studies (20, 25), and while the exact reasons for these differences remain unclear, varying approaches toward end-of-life and hospice care could have contributed to lower short-term mortality seen among blacks (26). Moreover, there was no statistically significant interaction between DTN and sociodemographic characteristics on the 30-day, 1-year, or 2-year mortality, indicating that all socio-demographic groups benefited equally from faster DTN times. The findings of our study further reinforce the need to implement specific interventions to reduce racial/ethnic disparities, improve stroke awareness and access to stroke healthcare among racial minorities (27).

This study reveals several other patient-related factors that affected the DTN times. Although women benefited from earlier treatment as much as men, fewer women received IVT within 60 min. This gender disparity has been shown in multiple other studies of IVT utilization (28, 29). A meta-analysis of 16 studies showed that women are less likely to receive IVT as compared to men. Although several sociocultural factors affecting women leading to delays in hospital arrival have been cited, these haven't been substantiated (30). Next, patients who received IVT within 60 min had a longer time from onset of symptoms to hospital arrival. A similar trend was shown in a large GWTG based study and it has been speculated that perhaps this may be related to a tendency of hospitals to take a more relaxed approach in patients who had a shorter onset of symptoms (31). Similarly, patients with atrial fibrillation were less likely to receive IVT within 60 min. One of the reasons for this may have been the additional time taken to obtain collateral clinical or laboratory information to establish IVT eligibility in these patients as many of these patients are expected to be on therapeutic anticoagulation.

The mechanisms by which IVT improves long-term mortality are not well-understood but better neurological function allowing increased physical activities, reduction in life-threatening pneumonia and increased independence leading to fewer medical complications have been cited among other reasons (32–35). In our study, the reduced rates of sICH in the patients with DTN ≤60 min may have contributed to reduced mortality in these patients as compared to the patients with DTN > 60 min.

Early reperfusion remains the cornerstone of success in AIS treatment which starts from early recognition of symptoms in the field. Delays in (1) recognition of symptoms, (2) access to medical care, and (3) initiation of treatment can ultimately negatively influence the final outcome of these patients. Moreover, transport *via* EMS with pre-hospital notification and single call stroke team activation has been shown to accelerate treatment times and promote favorable outcomes (36). This has been highlighted in our study with a higher proportion of patients transported *via* EMS having shorter DTN times. AIS treatment remains a complex process which requires close coordination and effective communication between various disciplines in the pre-hospital setting (EMS) as well as within the hospital (ED, Radiology, Nursing, Neurology, Laboratory, etc.). DTN time remains one of the most important modifiable variables in the treatment of AIS as the other variables are often influenced by several regional, socioeconomic and cultural characteristics of a community. This has led to DTN time being an important focus of several nationwide quality improvement initiatives in treatment of AIS (37).

The findings of our study are in keeping with previously published studies that showed faster treatment with IVT in larger hospitals as compared to small hospitals with lower annual IVT volume. Hospital-related reasons were previously shown to contribute to about 11% of patients getting delayed care leading to a substantial increase in DTN of more than 30 min in these patients (38). A recent study showed that with structural reorganization, critical training and well-defined protocol spearheaded by Emergency Physicians led to a significant reduction in DTN times (39). For hospitals with limited access to Vascular Neurology expertise, participation in Telestroke programs has also shown to reduce DTN times (40, 41). Thus, our study further emphasizes the need for each individual hospital to focus on continuous quality improvement to achieve safe and rapid reperfusion for eligible AIS patients. AHA/ASA recognizes this unmet need and suggests several strategies that hospitals can implement to improve their DTN for AIS patients (42). Despite strong recommendations, the rate of adoption of these strategies among different hospitals remains suboptimal (37). Furthermore, this study reinforces the compelling need to establish regional stroke systems of care focused on reducing pre-hospital and in-hospital delays in IVT.

The strengths of our study include the fact that it represents a large and diverse state-wide cohort of AIS patients treated with IV Alteplase in a real-world situation. A substantial proportion of our cohort included black patients who are often under-represented in population-based studies. There are currently no large registries that report data on longer-term outcomes of AIS after IVT, but with the unique ability to crosslink de-identified data from multiple resources, we have been able to provide reliable estimates of 30-day, 1-year, and 2-year mortality rates.

## Limitations

Our study has several limitations inherent to a retrospective analysis, such as unavailability of follow-up information on the patients with missing data leading to exclusion of such patients in the final analysis. As only GA state-based databases were used, patients who died out of state would not be captured in the analyses, potentially underestimating the rate of mortality. However, it is unlikely this would have any relation to DTN time and outcome. In addition, the mortality rates in our study are similar to previously published data suggesting the vast majority of death events occurred in GA and were captured in the state death records (11, 12). Our study only includes patients from the state of Georgia which suffers a high burden of stroke, so generalizability to other populations may be limited. Our study did not capture the cause of death or other confounding factors which can influence long-term mortality such as certain terminal diseases (cancer), previous alcohol consumption, or social characteristics such as marital status which could limit our ability to attribute reduction in mortality directly to faster DTN time. Lastly, there were some imbalances across the groups including higher rates of atrial fibrillation in the >60 min DTN patients which is known to be associated with worse functional outcomes and higher chances of SICH and mortality (43, 44). However, multivariable analyses adjusted for several potential confounders, including atrial fibrillation, and the relationship between DTN times and outcomes remained significant.

## Conclusions

This study of AIS patients across the state of Georgia provides robust evidence of 30-day, 1-year, and 2-year mortality benefit with faster IVT treatment. There was a consistent benefit of shorter DTN on mortality across all age, gender and race subgroups. It reinforces the critical need to expand and enhance quality improvement efforts at all stages of AIS treatment and for regions to establish systems of care to accelerate DTN times to reduce longer term mortality in these patients.

## Data Availability Statement

The raw data supporting the conclusions of this article will be made available by the authors upon reasonable request, without undue reservation.

## Ethics Statement

The studies involving human participants were reviewed and approved by Emory University. The Ethics Committee waived the requirement of written informed consent for participation.

## Author Contributions

NB, AB, and MF conceived the study. MI and RB provided statistical advice on study design and analyzed the data. NB and AB drafted the manuscript. MF takes responsibility for the paper as a whole. All authors contributed substantially to its revision.

## Conflict of Interest

RN reports consulting fees for advisory roles with Anaconda, Biogen, Cerenovus, Genentech, Imperative Care, Medtronic, Phenox, Prolong Pharmaceuticals, Stryker Neurovascular and stock options for advisory roles with Astrocyte, Brainomix, Cerebrotech, Ceretrieve, Corindus Vascular Robotics, Vesalio, Viz-AI, and Perfuze. The remaining authors declare that the research was conducted in the absence of any commercial or financial relationships that could be construed as a potential conflict of interest.

## Publisher's Note

All claims expressed in this article are solely those of the authors and do not necessarily represent those of their affiliated organizations, or those of the publisher, the editors and the reviewers. Any product that may be evaluated in this article, or claim that may be made by its manufacturer, is not guaranteed or endorsed by the publisher.
